# Corticofugal projection patterns of whisker sensorimotor cortex to the sensory trigeminal nuclei

**DOI:** 10.3389/fncir.2015.00053

**Published:** 2015-09-30

**Authors:** Jared B. Smith, Glenn D. R. Watson, Kevin D. Alloway, Cornelius Schwarz, Shubhodeep Chakrabarti

**Affiliations:** ^1^Department of Engineering Science and Mechanics, Pennsylvania State UniversityUniversity Park, PA, USA; ^2^Center for Neural Engineering, Huck Institute of Life Sciences, Pennsylvania State UniversityUniversity Park, PA, USA; ^3^Department of Neural and Behavioral Sciences, Pennsylvania State University College of MedicineHershey, PA, USA; ^4^Department of Cognitive Neurology, Hertie Institute for Clinical Brain Research, Eberhard Karls University of TübingenTübingen, Germany; ^5^Systems Neurophysiology, Werner Reichardt Center for Integrative Neurosciences, Eberhard Karls University of TübingenTübingen, Germany

**Keywords:** barrel cortex, corticofugal pathways, trigeminal nuclei, whisker, anterograde tracing, retrograde tracing

## Abstract

The primary (S1) and secondary (S2) somatosensory cortices project to several trigeminal sensory nuclei. One putative function of these corticofugal projections is the gating of sensory transmission through the trigeminal principal nucleus (Pr5), and some have proposed that S1 and S2 project differentially to the spinal trigeminal subnuclei, which have inhibitory circuits that could inhibit or disinhibit the output projections of Pr5. Very little, however, is known about the origin of sensorimotor corticofugal projections and their patterns of termination in the various trigeminal nuclei. We addressed this issue by injecting anterograde tracers in S1, S2 and primary motor (M1) cortices, and quantitatively characterizing the distribution of labeled terminals within the entire rostro-caudal chain of trigeminal sub-nuclei. We confirmed our anterograde tracing results by injecting retrograde tracers at various rostro-caudal levels within the trigeminal sensory nuclei to determine the position of retrogradely labeled cortical cells with respect to S1 barrel cortex. Our results demonstrate that S1 and S2 projections terminate in largely overlapping regions but show some significant differences. Whereas S1 projection terminals tend to cluster within the principal trigeminal (Pr5), caudal spinal trigeminal interpolaris (Sp5ic), and the dorsal spinal trigeminal caudalis (Sp5c), S2 projection terminals are distributed in a continuum across all trigeminal nuclei. Contrary to the view that sensory gating could be mediated by differential activation of inhibitory interconnections between the spinal trigeminal subnuclei, we observed that projections from S1 and S2 are largely overlapping in these subnuclei despite the differences noted earlier.

## Introduction

The trigeminal sensory nuclei constitute the first synaptic station of sensory processing in the whisker-to-barrel pathway conveying tactile information from the mystacial vibrissae to the primary somatosensory cortex (S1). The trigeminal sensory nuclei consist of the nucleus principalis (Pr5), and the spinal nuclei—oralis (Sp5o), interpolaris (Sp5i) and caudalis (Sp5c). In addition to projection neurons that ascend the neuraxis in four distinct sensory pathways (Lo et al., 1999; Veinante and Deschênes, 1999; Pierret et al., 2000; Veinante et al., 2000a; Furuta et al., 2009; Wimmer et al., 2010; Ohno et al., 2012), the trigeminal sensory nuclei also contain an intricate network of interneurons that interconnect the trigeminal nuclei and have been implicated in shaping trigeminal receptive fields (Timofeeva et al., 2004; Furuta et al., 2008; Bellavance et al., 2010). Based on the relative proportion of interneurons, recent work has shown that Sp5i can be subdivided into a rostral area (Sp5ir) composed mainly of excitatory multi-whisker neurons that project to the thalamic posterior medial nucleus (POm) and a caudal area (Sp5ic) comprised mainly of single whisker inhibitory interneurons (Furuta et al., 2006, 2008). The trigeminal sensory nuclei also receive descending projections from S1 and the secondary somatosensory cortex (S2; Wise and Jones, 1977; Killackey et al., 1989; Jacquin et al., 1990; Furuta et al., 2010).

Sensory responses to active touch (i.e., moving whiskers) in both thalamus and cortex have smaller amplitudes than passive touch responses (Fanselow and Nicolelis, 1999; Hentschke et al., 2006; Ferezou et al., 2007; Lee et al., 2008). Presumably, this difference is present in Pr5 and occurs as a result of intra-trigeminal inhibitory processes (Lee et al., 2008). A recent study based on electrical cortical stimulation suggested that corticofugal projections alter the trigeminal response profiles by selectively gating the ascending lemniscal pathway during whisking and non-whisking conditions (Furuta et al., 2010). According to this hypothesis, descending corticofugal projections from S1 barrel cortex and S2 exert differential effects on the neurons in Pr5 that give rise to the lemniscal pathway. While S2 projections are thought to innervate inhibitory interneurons in Sp5ic that project to Pr5 to close the sensory gate, S1 septal columns presumably project to Sp5c interneurons, which, via Sp5ic interneurons, disinhibit neurons in Pr5, to open the lemniscal gate (Furuta et al., 2010). This hypothesis rests on the specificity of S1 and S2 projections to the sensory trigeminal nuclei and suggests selective innervation of Sp5c by S1 septal columns and Sp5ic by S2. However, anatomical data on the precise origin of corticofugal projections to different trigeminal nuclei and their pattern of termination have never been quantified.

To address this issue, we used anterograde and retrograde tracers to quantify the total number of trigeminal corticofugal terminals, their areal distribution, and their origin by cortical area, including S1 barrel or septal columns. Our results show similar patterns of corticofugal projections from S1 and S2 with overlapping terminal arbors in both Sp5ic and Sp5c. However, we also report some important differences in their respective areal extents especially in their three dimensional geometry.

## Materials and Methods

Neuronal tracer injections were performed in female (180–350g) and male (240–450g) Sprague Dawley rats. Injections performed in the United States of America conformed to NIH guidelines and were approved by the Penn State University Institutional Animal Care and Use Committee. Injections performed in the Federal Republic of Germany complied with German Federal Law and were done in accordance with the policy on the use of animals in neuroscience research of the Society for Neuroscience.

### Animal Preparation and Surgery

Rats were anesthetized with an initial IP or IM injection of ketamine HCl (40–100 mg/kg) and xylazine (10–12mg/kg), placed in a stereotaxic frame (David Kopf Instruments, Tujunga, CA, USA) and subsequently maintained in an anesthetized state with 0.5–2% isoflurane (1-Chloro-2,2,2-trifluoroethyl-difluoromethylether) in medical oxygen. The anesthetic state of the animal was continuously tested by monitoring the hindpaw withdrawal reflex and the isoflurane adjusted so as to ensure the complete absence of such responses. Body temperature, monitored using a rectal probe, was maintained at 37°C using a homeothermic blanket (Harvard Apparatus, MA, USA) and ophthalmic ointment applied to the cornea to prevent drying. A 2% solution of mepivicaine or xylocaine was injected under the scalp for local anesthesia, and a midline incision was used to expose the skull.

Craniotomies were made over the region of interest, including Sp5ir (12.5–13.0 mm caudal, 2.0–3.0 mm lateral), Sp5ic (13.5–14.0 mm caudal, 2.0–3.0 mm lateral), S1 (0–4.0 mm caudal, 3.0–7.0 mm lateral), S2 (0–4.0 mm caudal, 6.0–9.0 mm lateral) and M1 (1.0–3.0 mm rostral, 0.5–2.5 mm lateral) using published co-ordinates (Paxinos and Watson, 2006). Following the tracer injections, the incision was closed using surgical sutures, an antibiotic ointment containing sodium fusidate was applied to the wound, and the animals received a subcutaneous injection of the anti-inflammatory analgesic carprofen or dexamethasone (5 mg/kg). Animals underwent a 10 day post surgical survival period. Animals were administered the antibiotic enrofloxacin (2.5 mg/kg) either as a postsurgical IM injection or by adding the antibiotic to the drinking water provided.

### Electrophysiology and Mapping

To identify the whisker representations in S1, S2 and Sp5i, a mapping electrode with an impedance between 1 and 3 MΩ was lowered either into layer IV of cortex (400–800 μm deep) to access S1 and S2, or 6–7 mm deep below the cerebellar surface to access the Sp5i whisker representations, respectively. Extracellular neural discharges were amplified using an extracellular amplifier (Dagan 2200; Dagan Corp., Minneapolis, MN, USA or ME64-FAI-MPA system, Multichannel Systems, Reutlingen, Germany) with a gain of 20–50k, bandpass filtered between 300–20,000 Hz and monitored on an oscilloscope and an audio monitor.

The contralateral whiskers, in case of cortical recordings, or the ipsilateral whiskers, in case of brainstem recordings, were deflected using a wooden rod to ascertain the receptive field properties of the neuronal signals. For S2 mapping, the electrode was moved laterally from S1 until a reversal of the whisker map was encountered, which was taken to be the S1, S2 boundary. For brainstem recordings, the electrode passed through whisker responsive regions in the cerebellum (2–3 mm deep) followed by non-whisker responsive regions before entering the brainstem trigeminal whisker representations (6–7 mm deep). To identify M1 whisker cortex, saline-filled pipettes were inserted orthogonally into the brain at a depth of ~1.5 mm, and trains of cathodal pulses (0.7 ms pulse width) were administered at 250 Hz for ~80 ms at a current level of 10–100 μA, while observing the contralateral whisker pad for whisker twitches. Injections were centered around the representation of the caudal whiskers of rows C, D and E in all cases. After identifying the whisker representations in S1, S2, M1 or Sp5i, tracer injections were made in these regions.

### Tracer Injections

The anterograde tracers used were a 15% solution of biotinylated dextran amine (BDA, Invitrogen) or a 15% solution of Fluoro-Ruby (FR, Invitrogen) in 0.01M phosphate buffered saline (PBS). A 2% solution of Fluorogold (FG, Fluorochrome, Denver, CO, USA) in saline was employed as a retrograde tracer. BDA was pressure injected through a glass pipette cemented onto the tip of a Hamilton syringe (150 nl per injection site, 2–3 injections per region) whereas FG or FR were injected iontophoretically by passing a current of 2.5–5.0 μA for FG and 11 μA for FR for 10–20 min using a 7 s duty cycle. The most caudal injections into the brainstem succeeded in labeling Sp5ic, but not Sp5c, which was inaccessible because it is located under thick neck musculature. All brainstem tracer deposits consisted of injections made at 2–3 depths separated by ~200 μm to ensure filling of the complete nucleus.

### Perfusion and Histology

Following a 10 day survival period, animals were deeply anesthetized with sodium pentobarbital (50–100 mg/kg) until pain reflexes were absent. Each rat was transcardially perfused with isotonic saline followed by 4% paraformaldehyde and 4% paraformaldehyde containing 10% sucrose. The brain was removed, cortical slabs were prepared as described before (Chakrabarti and Alloway, 2006; Smith and Alloway, 2013) and the tissue stored in 4% paraformaldehyde with 30% sucrose at 4°C for 2–3 days before being sectioned using a freezing microtome at a thickness of 60 μm. The cortex was removed from the underlying sub-cortical tissue, flattened between two glass slides and then sectioned horizontally. The sections corresponding to layer IV were processed for cytochrome oxidase (CO) as described previously (Wong-Riley, 1979; Land and Simons, 1985; Smith and Alloway, 2013), whereas the remaining sections were processed to visualize the tracers. The brainstem was removed by making two cuts, one in a coronal plane at the level of the cerebral aqueduct and the other in a horizontal plane just below the cerebellum. The brainstem was then sectioned horizontally, and alternate sections were processed for CO or the tracer. In two cases receiving retrograde brainstem injections, the cortex was sectioned coronally to determine the laminar location of retrogradely labeled cells. In another three cases receiving anterograde injections in S1 and M1, the entire brain was sectioned coronally to determine the differences in S1 and M1 projections to various trigeminal and other brainstem nuclei.

For FG and FR visualization, sections were mounted on gelatinized slides and coverslipped. For BDA visualization, sections were processed as described previously (Smith et al., 2012a). Briefly, sections were rinsed in 0.3% H_2_O_2_ to quench background staining, incubated in 0.3% Triton X-100 in 0.1M PBS before being incubated for 2 h in an avidin-biotin horseradish peroxidase solution (Vector Novocostra Laboratories, Burlinghame, CA, USA) in 0.3% TritonX-100 in 0.1M PBS. Following incubation, sections were rinsed twice in 0.1M PBS and then incubated in 0.06% diaminobenzidine (DAB) containing 0.0005% H_2_O_2_, 0.05% NiCl_2_ and 0.02% CoCl_2_ in 0.1M tris buffer (*pH* = 7.2) for 10 min. The DAB reaction was stopped by washing in 0.1M PBS and the sections were mounted, dried overnight and coverslipped.

### Definition of Trigeminal Sensory Nuclei

**Figure 1:**
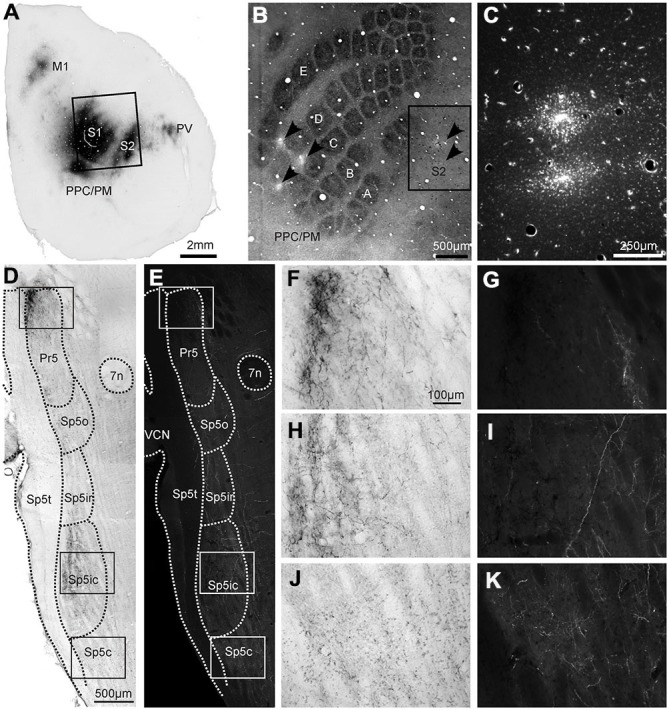
**Labeling patterns in the trigeminal sensory nuclei following dual anterograde tracer deposits in the whisker representations of primary (S1) and secondary somatosensory (S2) cortices. (A)** Photomicrograph of tangential cortical section processed for Biotinylated Dextran Amine (BDA) **(B)**. Locations of the tracer deposits in the **(C,D)** rows of S1 (BDA) and **(B–D)** rows of S2 (FR) shown on a tangential section of the cortex through layer IV, processed for cytochrome oxidase (CO). **(C)** Adjacent section processed for the fluorescent tracer Fluoro Ruby (FR), which was injected at two locations in the S2 whisker representation corresponding to the inset in **(B)**. **(D)** Horizontal section through the contralateral trigeminal sensory nuclei processed for BDA shows different nuclei and anatomical landmarks (dotted lines), identified from an adjoining CO section. **(E)** The same section viewed using a TRITC filter for FR visualization showing labeled terminals across trigeminal sensory nuclei. **(F,H,J)** Photomicrographs of the areas in the insets of 1D show the morphology of BDA-labeled terminals in Pr5, Sp5ic and Sp5c, respectively. **(G,I,K)** The areas under the same insets **(E)** but viewed using a TRITC filter to show the morphology of FR labeled terminals. The BDA reaction product can be seen on the same photomicrographs. Abbreviations: M1, primary motor cortex; PPC/PM, posterior parietal cortex/posterio-medial cortex; Pr5, principal trigeminal nucleus; Sp5o, spinal trigeminal nucleus pars oralis; Sp5ir, spinal trigeminal nucleus pars interpolaris rostral; Sp5ic, spinal trigeminal nucleus pars interpolaris caudal; Sp5c, spinal trigeminal nucleus pars caudalis; Sp5t, spinal trigeminal tract; 7n, nucleus of the 7th cranial nerve; VCN, ventral cochlear nucleus.

The boundaries of the various trigeminal sensory nuclei were defined in alternate sections processed for CO visualization. The Sp5ic-Sp5c boundary was observed in all sections as a clear transition between the densely stained Sp5i and the lightly stained Sp5c neuropil in the CO sections at the level of the obex at the location of the newly discovered pars muralis nucleus (Matthews et al., 2015). The Sp5ic-Sp5ir boundary was defined using a transition from uniform staining in Sp5ir to CO labeled patches observed in Sp5ic. This coincided in most cases with the lateral bulge observed in the spinal trigeminal tract which imparts the Sp5i nucleus its characteristic teardrop shape. The Sp5o-Sp5ir boundary was the most difficult to demarcate as there were no clear transitions in CO labeling. Therefore the caudal edge of the ventral cochlear nucleus (VCN) was defined as the caudal boundary of Sp5o in keeping with earlier conventions (Furuta et al., 2006). Finally, Pr5 was defined as extending from the rostral boundary of intense CO labeling to the caudal edge of the nucleus of the 7th cranial nerve (7n). Finally the boundaries were drawn by two independent observers and averages across the two determined as the true boundary. Examples of boundaries can be seen in Figure 1.

### Anatomical Analysis

An Olympus BH-2 microscope (Olympus, Miami, FL, USA) and a Axio Imager Z2 (Carl Zeiss Microscopy, Jena, Germany) equipped for fluorescent microscopy were used to acquire images of the histological sections. Terminals labeled with BDA were viewed in brightfield, terminals labeled with FR were seen with a TRITC filter (41002, Chroma Technologies, Bellows Falls, VT, USA, emission: 570–650 nm; excitation: 510–560 nm) and FG-labeled terminals were viewed with a Brightline HC excitation filter (F39–377, AHF AG, Tübingen, Germany, excitation: 200–399 nm) in combination with a highpass emission filter (F76–516, AHF AG, Tübingen, Germany, emission: 529–900 nm). Axonal varicosities, representing en passant synapses (Voigt et al., 1993; Kincaid and Wilson, 1996; Meng et al., 2004), were plotted using an Accustage plotting system (Accustage, St. Paul, MN, USA) consisting of a XY digitizer attached to the microscope stage. Cortical slices containing retrogradely labeled cells were photographed using Axiovision software with the MosaicX function to obtain high magnification (20X) photomicrograph montages of the entire section. Images were converted into grayscale, the retrogradely labeled cells were extracted from background using a binary threshold for labeling intensity to define labeled somata (Image J software) and their positions marked. Different cortical sections were aligned using blood vessels running orthogonal to the surface as alignment control points.

All plotted reconstructions, which contain plots of varicosities, retrogradely-labeled cells, and section outlines were transferred to the MATLAB (Mathworks, Natick, MA, USA, Ver. 2013b) environment where further analyses were performed. To quantify anterograde labeling in brainstem trigeminal sensory nuclei, each plotted section was loaded and aligned with the sections from other dorsoventral levels using a simple affine image transformation (Image Processing Toolbox). To quantify labeling in different nuclei, all labeled terminals within the respective nuclear boundaries were counted. To obtain three dimensional distributions (Figure 4), labeled terminals were counted in 200 μm rostro-caudal bins for each section (dorsoventral level, section thickness: 60 μm), aligned to the rostral border of Pr5 for each section, and displayed using a 3D surface plot. Dorsal sections where nuclear boundaries could not be discerned from the CO sections were not included (black rows). To obtain rostro-caudal distributions of labeled terminals (Figure 5), each trigeminal sensory nucleus was binned using 10 equal bins and the varicosity count calculated. All terminal counts were normalized by the total number of terminals resulting from that particular injection to account for differences in tracer volumes injected into different cortical areas.

To compute overlap between the terminals from S1 and S2 in the different trigeminal sensory nuclei, each nucleus was binned using 50 μm bins and a two dimensional histogram of binned varicosities in each of these bins for S1 and S2 terminals computed separately. Those bins which contained positive values for both S1 and S2 terminals, i.e., those bins with at least one labeled terminal from S1 and S2 were then expressed as a percentage of the total number of bins containing labeled terminals in that nucleus.

For retrograde tracer injections, the layer IV cortical sections, processed for CO, and the other sections processed for visualization of the tracer were aligned by using the blood vessels running orthogonal to the cortical surface as control points. The outlines of the S1 barrel cortex and S2 as well as of S1 barrels were then overlaid on the sections containing retrogradely labeled cells and the total number of such cells were counted in each compartment. Total number of retrogradely labeled cells across all sections in each region was then divided by the total area of that particular region to yield cell count densities.

## Results

A total of six male and eight female Sprague Dawley rats were used for this study. Of the males, three received dual tracer injections of BDA and FR into S1 and S2 whisker representations, respectively. The remaining three male rats were from a previous study looking at forebrain connectivity (Smith et al., 2012b), which had received BDA and FR injections into S1 and M1 whisker representations. Of eight female rats, all received FG injections into different trigeminal sensory nuclei. FG was placed in Sp5ic (*n* = 2) the entire Sp5i nucleus (*n* = 4), or in Sp5ir and the Sp5ic/Sp5c boundary (1 each). Cases entered the present data set only when the tracer injection could be readily identified and localized, was confined to the trigeminal nuclei and did not encroach upon either the trigeminal tract or other brainstem nuclei such as the reticular formation, and produced retrograde labeling in cortex.

### Anterograde Tracing: Extent of Corticofugal Projections in Trigeminal Sensory Nuclei

An example of labeling patterns in the trigeminal nuclei following dual tracer injections in the whisker regions of S1 and S2 is shown in Figure 1. In this case, BDA was injected into the C and D rows of the S1 barrel field at three separate sites (Figure 1B) whereas two separate FR tracer deposits were placed in the S2 C and D row representations (Figure 1C). Injections into the S1 barrel cortex produced labeling in various cortical areas such as M1, S2, the posterior parietal or posterio-medial cortex (PPC/PM; Carvell and Simons, 1986; Koralek et al., 1990; Kim and Ebner, 1999; Smith and Alloway, 2013) as well as the parietal ventral region (PV; Krubitzer et al., 1986; Fabri and Burton, 1991), as reported earlier. Labeled terminals for both BDA and FR were distributed contralaterally across all four sensory trigeminal nuclei i.e., Pr5, Sp5o, Sp5i and Sp5c (Figures 1D,E), as reported earlier (Jacquin et al., 1990; Desbois et al., 1999; Aronoff et al., 2010; Haque et al., 2012; Tomita et al., 2012; Takatoh et al., 2013; Sreenivasan et al., 2014). Sections stained for CO immunohistochemistry could be used for the definition of the trigeminal nuclear boundaries which were then superimposed on the sections processed for tracer visualization (Figures 1D,E; see “Materials and Method” Section for details). Overall anterograde labeling patterns were similar for S1 and S2 injections. Specifically Pr5 and Sp5ic were densely innervated by corticofugal projections originating from both S1 and S2. However, there were also important differences as seen when comparing Figures 1D,E, namely that Sp5o, Sp5ir and Sp5c all contained more FR labeled terminals (S2) than BDA labeled ones. The difference in the amount of terminal labeling resulting from the two tracers in Sp5c is apparent when comparing Figures 1J,K.

**Figure 2:**
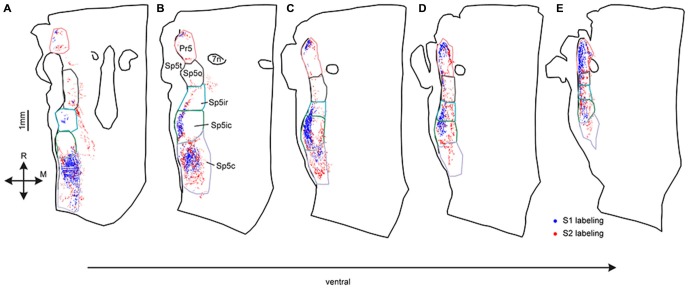
**Digital reconstructions of anterogradely-labeled terminals in five horizontal sections spanning the entire dorsoventral extent of the trigeminal sensory nuclei.** Labeled S1 corticofugal terminals are shown in blue, S2 terminals in red. Sections are arranged from the most dorsal **(A)** to the most ventral **(E)**. Outlines of the different trigeminal sensory nuclei, defined using adjacent CO sections are shown superimposed on the plotted terminals. Consecutive sections were 180 μm apart.

These differences between S1 and S2 corticofugal labeling patterns were best visualized when examining trigeminal brainstem horizontal sections at different dorso-ventral levels. As seen in Figures 2A,B, at more dorsal levels, the labeled terminals from both S1 and S2 were distributed in discrete patches centered in Pr5, Sp5ic and Sp5c. These patches of labeling were mostly separated by zones of little or no labeling in between them. The S1 terminals were distributed in three separate clusters located in the Pr5, Sp5ic and Sp5c nuclei whereas the S2 terminals also innervated the Sp5o nucleus. In ventral sections, S1 labeling in Sp5c became scant with dense labeling in Pr5 and Sp5ic, and some labeled terminals in Sp5ir (Figure 2C), whereas the labeled projection terminals from S2 were more evenly distributed across all of the different trigeminal sensory nuclei (Figures 2C–E). Thus, Pr5, Sp5ic, Sp5c, received both S1 and S2 projections whereas Sp5o and Sp5ir received mainly S2 projections. Labeling in Pr5 frequently exhibited a discrete, mirrored pattern with S1 terminating in a lateral patch and S2 terminating in a medial patch. Corticofugal projections to Sp5c had a distinct dorso-ventral gradient with most S1 projections targeting dorsal Sp5c whereas S2 projections spanned the entire dorso-ventral extent of the nucleus.

**Figure 3:**
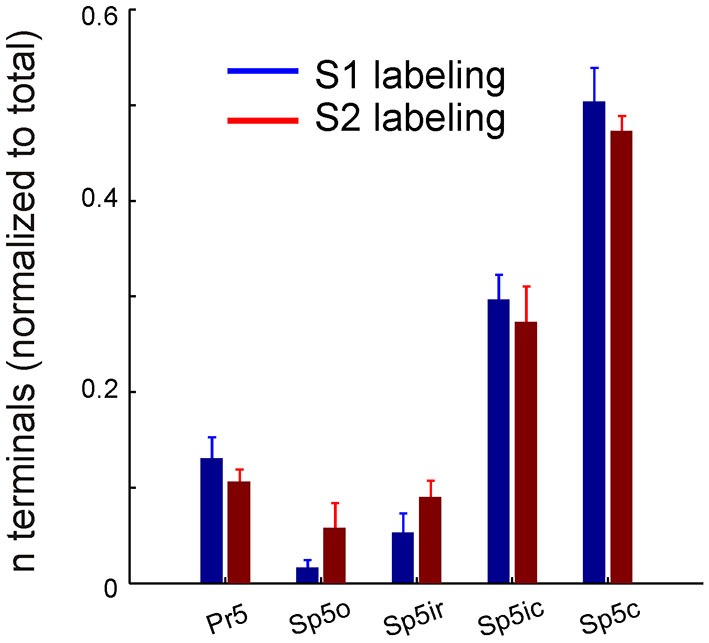
**Corticofugal terminal counts in the trigeminal sensory nuclei following dual tracer injections in S1 and S2.** Bar graph showing the total number of labeled terminals resulting from anterograde tracer injections into S1 (blue) and S2 (red) whisker representations, normalized by the total number of terminals resulting from each injection, averaged across animals (*n* = 3). Brackets indicate standard error of the mean.

We next counted the number of labeled terminals originating from S1 and S2 in the various trigeminal sensory nuclei after normalizing each by the total number of terminals resulting from that particular injection (Figure 3). The overall similar pattern of S1 and S2 labeling across the different nuclei was reflected in these counts which showed similar distributions, with the highest number of corticofugal projections terminating in Sp5c, Sp5ic and Pr5, in that order. A one way ANOVA showed that this difference across nuclei was statistically significant for both S1 (*F* = 70.22; *p* < 10e^−6^) and S2 terminals (*F* = 55.11; *p* < 10e^−6^). Within each nucleus, however, there were no significant differences between S1 and S2 terminal counts.

Statistical analysis failed to reveal differences in the quantity of labeled terminals within overall volume of the trigeminal nuclei, but we observed dorsal-ventral differences in the projections from S1 and S2. (Figure 2). For example, although Sp5c contained the highest number of S1 terminals amongst all the trigeminal sensory nuclei, the S1 projections were restricted to the dorsal aspect of the nucleus whereas the S2 projections were distributed throughout (Figure 2). For a more accurate depiction of the pattern of corticofugal labeling we analyzed the three dimensional distribution of labeling across nuclei with respect to the rostro-caudal and dorso-ventral dimensions in the brainstem.

### Three Dimensional Distribution Uncovers Unique Projection Patterns from S1 and S2

**Figure 4:**
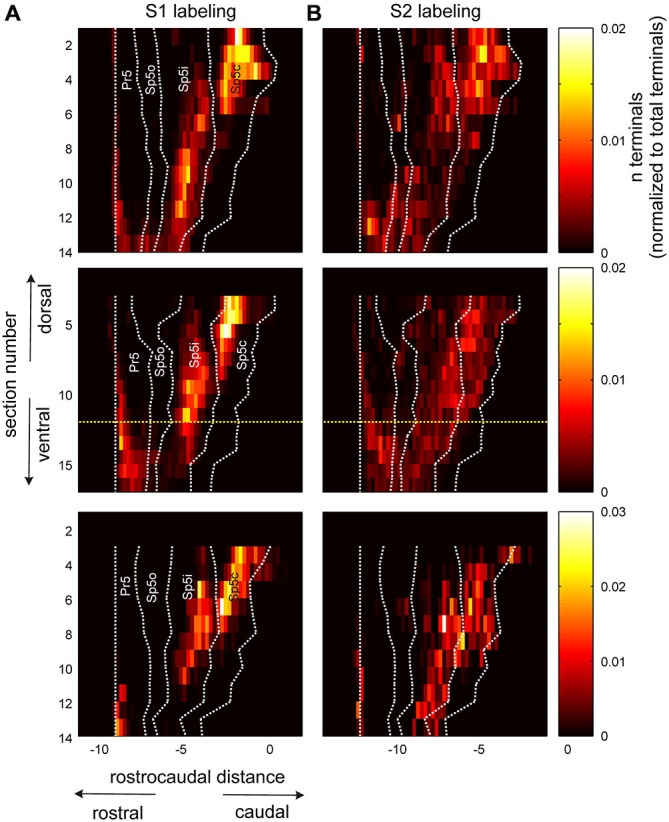
**Pattern of corticofugal terminal distribution across trigeminal sensory nuclei in both dorsoventral and rostro-caudal dimensions. (A)** Terminal counts from an anterograde tracer injection into S1 cortex, normalized by total S1 terminal counts tallied across all horizontal sections through the brainstem. The different horizontal sections are arranged along the *y* axis with the dorsal most section located at the top. The rostral and caudal boundaries of the different trigeminal sensory nuclei have been overlaid on top using dotted white lines. All rostrocaudal measurements were normalized to the rostral boundary of the Pr5 nucleus for each section. Three animals are represented in the three rows. The yellow dotted lines in the second row denote the section shown in Figure 1D. **(B)** Identical plots for terminals labeled with an anterograde tracer injected into S2. All rostrocaudal distances are in mm.

The apparent disparity between individual labeling patterns (Figure 2) and the quantification of distributions (Figure 3) was resolved by quantifying the terminal counts from S1 and S2 in three dimensional space. Figure 4 shows the quantification of normalized terminal counts obtained by binning them in 200 μm bins for each dorso-ventral section (see “Materials and Method” Section for details) for all three animals for S1 and S2 labeled terminals separately. The rostro-caudal boundaries of each nucleus at each dorso-ventral level were overlaid on the terminal binned counts and all rostro-caudal measurements normalized to the rostral-most point of the Pr5 nucleus for each section.

The similarity of S1 and S2 labeling patterns is apparent upon comparing panels A and B in Figure 4. Both cortical areas targeted all trigeminal sensory nuclei, as shown before, with labeling in Sp5o and caudal Pr5 being sparse for both cortical regions across animals. However, unlike earlier depictions, there were important differences in the dorso-ventral and rostro-caudal extents and patterns of labeling resulting from S1 and S2 tracer injections. The projections from S2 terminated in a continuous distribution that extended from dorsal Sp5c to Sp5ir with labeled terminals in Sp5c at almost all dorso-ventral levels. By comparison, S1 labeling formed discrete clusters or patches centered in dorsal Sp5c and ventral Sp5ic with a clear break between these two patches at the Sp5ic/Sp5c border. S1 labeling in Sp5c occurred more dorsally across animals and ventral Sp5c remained largely deficient of S1 terminals. S2 labeling targeted Sp5ir and Sp5o with large numbers of labeled terminals in these two nuclei observed in at least 2 of the 3 animals. By contrast Sp5o was largely devoid of S1 projections except in the most ventral sections. Finally, the corticofugal labeling in Pr5 tended to be concentrated at the rostral aspect of the nucleus except in very ventral sections.

It is worthwhile to point out the very low inter-animal variability of corticofugal labeling in the trigeminal sensory nuclei as seen in Figure 4. The labeling patterns across dorso-ventral and rostro-caudal dimensions are extremely similar across animals including the trough in S1 terminal count separating Sp5ic and Sp5c across the different dimensions. The fairly abrupt decrease in S1 labeling in Sp5ir and Sp5o is also remarkably conserved across animals.

**Figure 5:**
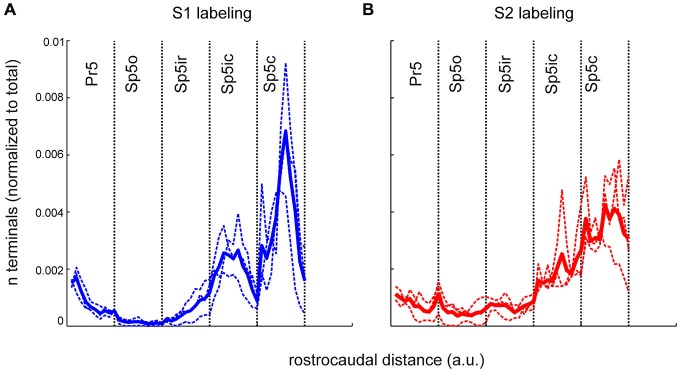
**The rostrocaudal distribution of labeled terminals from S1 and S2 cortical injections across the different trigeminal sensory nuclei. (A)** The binned terminal counts resulting from an anterograde injection into the S1 cortex of three rats. Each nucleus, irrespective of its actual rostrocaudal extent, has been binned into 10, equally spaced bins. The width of each bin varies according the rostro-caudal extent of each nucleus with Pr5 bins being the smallest. Dotted lines show the binned terminal counts averaged across all sections, for each animal. Thick line denotes the mean of these averages across animals. **(B)** Identical plot of terminal distribution from the S2 injection. a.u., arbitrary units.

To represent the differences in S1 and S2 labeling within each nucleus, terminal counts within each nucleus were binned using 10 equally spaced rostro-caudal bins and are shown in Figure 5. Thus, the rostro-caudal differences within each individual nucleus could be separately represented instead of being grouped together as in Figure 3. The clustered nature of S1 projection patterns, seen in Figures 2, 2, 4, was apparent using this binning procedure and the S1 terminal counts had a tri-modal rostro-caudal distribution with peaks occurring in rostral Pr5, Sp5ic and Sp5c with a deep trough separating the latter two peaks (Figure 5A). By contrast, S2 terminals showed a unimodal distribution with a peak located in the Sp5c. The relative low inter-animal variability is again apparent when comparing the individual animals with the respective group averages.

We also computed the overlap between the anterogradely labeled terminals from S1 and S2 across the different trigeminal sensory nuclei using 50 μm bins (data not shown). There was an overlap of 10–20% between S1 and S2 terminals in all the different trigeminal nuclei, and there was no significant difference between any of the different nuclei. Therefore, despite the differences in the number of terminals originating from S1 and S2 in the different nuclei, the spatial overlap between these terminals was unchanged across nuclei.

### Retrograde Tracing: Corticofugal Projections Arise from Both Barrels and Septa

**Figure 6:**
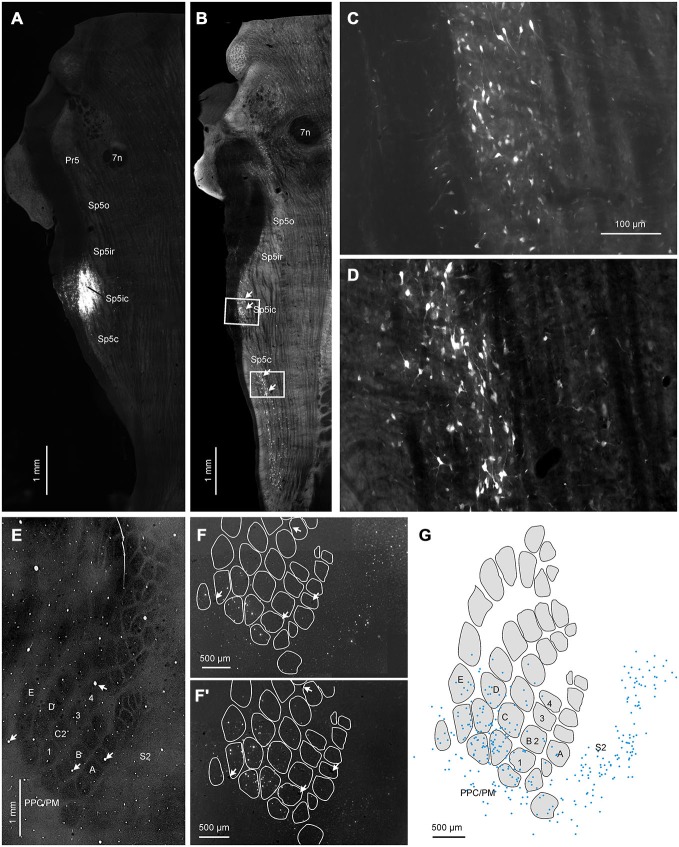
**Representative example showing cortical labeling patterns following a retrograde tracer (fluorogold, FG) deposit into the Sp5ic nucleus. (A)** Horizontal section through brainstem showing location of the FG injection site. **(B)** Adjacent horizontal section showing the presence of clusters of retrogradely labeled cells (arrows) located in Sp5ic and Sp5c. White boxes denote insets shown at greater magnification in panels **(C–D)**. **(E)** Horizontal section through layer IV of the contralateral S1 cortex visualized for CO staining showing the spatial extents of the S1 barrel field, S2 and PPM/PC. **(F,F′)**. Two horizontal sections through infragranular layers, processed for visualization of the tracer, showing retrogradely labeled cells in S1, S2 and PPC/PM. **(G)** Digital reconstructions of the positions of the retrogradely labeled cells shown superimposed upon outlines of layer IV barrels and inter-barrel septa obtained from the CO-stained section. The sections containing the CO stained barrel field and the retrogradely labeled cells were aligned using blood vessels running orthogonal to the cortical surface (panels **(E,F,F′)** arrows) as control points.

To complement our anterograde tracing data, we injected FG into various trigeminal nuclei at different rostro-caudal levels in eight rats. Figure 6 shows a representative example where the FG deposit was placed in the Sp5ic nucleus (Figure 6A). As reported before Furuta et al. (2010), one of the largest sources of projections to the trigeminal nuclei are other trigeminal nuclei and we observed a dense strip of retrogradely labeled cells stretching caudally from the injection site (Figure 6B). Retrogradely cells were found within the Sp5ic itself (Figure 6C) and in the Sp5c (Figure 6D). In cortex, retrogradely labeled cells were distributed across the S1 barrel cortex (Figures 6F,F′) spanning both barrel and septal columns (Figure 6G) as well as S2 and the PPC/PM (Figures 6F,F′). Barrel and septal columns were defined as regions in supra- and infragranular layers vertically aligned with layer IV barrels and septa respectively (Alloway et al., 2004; Chakrabarti and Alloway, 2006). Retrogradely labeled cells were located exclusively in the infragranular layers (layers V/VI). It is noteworthy to mention that there was a complete absence of retrogradely labeled cells within the M1 vibrissal representation as defined by stereotaxic coordinates. This is in accordance with previous reports (Miyashita et al., 1994; Urbain and Deschênes, 2007).

Data across four animals that received tracer injections into either the entire Sp5i nucleus or Sp5ic and in which the cortex was sectioned tangentially were pooled together to quantify the number of retrogradely labeled cells located in barrel and septal columns respectively. These animals were chosen because Sp5ic receives both S1 and S2 projections and injections covering this nucleus would enable a quantification of projection neurons in both S1 and S2 in the same animal. S1 barrel cortex contained 55% of the labeled cells whereas the S2 cortex contained 29% (data not shown). The remaining 16% of retrogradely labeled cells were located in the dysgranular zone or the PPC/PM. When the cell counts for the S1 barrel and septal columns were separately computed, the barrel and septal columns accounted for 30 and 25% of the labeled cells, respectively.

We also computed the cell density by dividing the total cell count by the area of the barrel, septal or S2 regions. Barrel, septal and S2 compartments had average cell densities of 30, 26 and 23 cells/mm^2^ respectively and a one way ANOVA failed to show any statistical significance in cell densities across these different regions (*n* = 4, *F* = 0.07, *p* = 0.93). Therefore the somata of neurons giving rise to the corticofugal projections to Sp5i, which is one of the major recipients of cortical projections to the trigeminal sensory nuclei, are approximately equally distributed over S1 and S2 and the barrel and septal columns.

### Retrograde Tracing Confirms Differences between S1 and S2 Corticofugal Terminal Distribution

**Figure 7:**
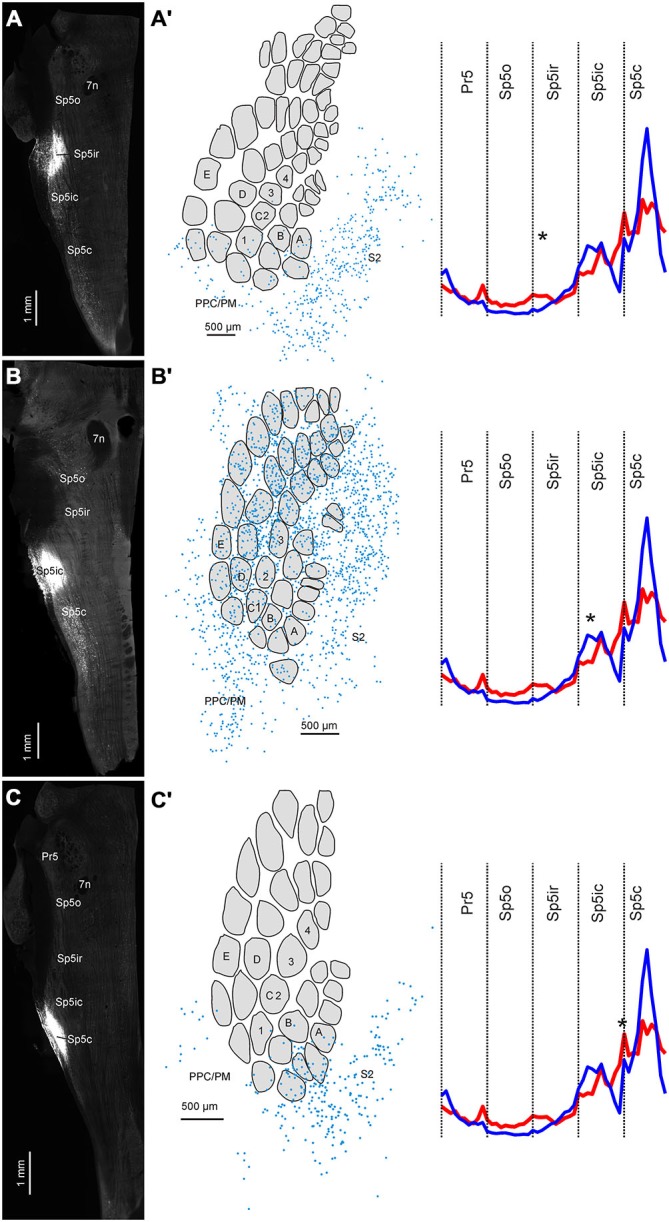
**Three cases showing the distribution of retrogradely labeled cells in cortex following FG deposits at different rostrocaudal positions in the trigeminal sensory nuclei. (A)** FG deposit into the Sp5ir. **(A′)** Digital reconstruction of retrogradely labeled cells in cortex superimposed on an outline of layer IV barrels and septa. Schematic shows the average distribution of binned terminals across the different trigeminal sensory nuclei (taken from Figure 5) with the location of the current injection marked with an asterisk. **(B–C′)** Identical plots for two other cases receiving tracer deposits into the Sp5ic and the Sp5ic/Sp5c boundary.

We injected the ventral depths of Sp5ir and the Sp5ic/Sp5c boundary of two animals to test whether the pattern of corticofugal projections revealed by our anterograde tracing experiments were confirmed by retrograde labeling. Data from these two animals along with a third case that received FG in Sp5ic are shown in Figure 7. The injections in Figures 7A–C targeted the Sp5ir, Sp5ic and Sp5ic/Sp5c boundary respectively. As shown earlier in Figure 6, every injection resulted in retrogradely labeled cells in the Sp5ic and Sp5c. These intra-trigeminal projection neurons have been reported before and have been shown to be primarily inhibitory interneurons (Avendaño et al., 2005; Furuta et al., 2010; Martin et al., 2014).

When the positions of retrogradely labeled cells in cortex were determined relative to the S1 barrel field, labeling patterns across S1 and S2 confirmed the results from our anterograde tracer injections. An injection into Sp5ir resulted in a majority of labeled cells in S2 and only a minority in S1 and PPC/PM (Figures 7A,A′). This is consistent with anterograde data suggesting that Sp5ir mainly receives corticofugal projections from S2 (Figures 2–5). A cartoon showing the average rostro-caudal labeling across animals resulting from anterograde injections (Figure 5) with an asterisk marking the site of the current retrograde injection is replotted to the right to allow direct comparison of the anterograde and retrograde data.

A FG deposit further caudal in Sp5ic, resulted in a large number of retrogradely labeled cells across S1, S2 and PPC/PM (Figures 7B,B′). The site of this injection corresponded with one of the peaks in the trimodal anterograde labeling curve obtained for S1 corticofugal terminals.

An injection of FG further caudal centered around the Sp5ic/Sp5c boundary again resulted in a majority of retrogradely labeled cells in S2 (Figures 7C,C′). The site of this last injection corresponded to the area marked by a lack of S1 terminals as shown in the average S1 corticofugal terminal distribution trace obtained earlier from anterograde labeling. Although this curve shows a second peak in S1 labeling in Sp5c which was also covered by this injection, this Sp5c peak in S1 labeling is located more dorsally than the Sp5ic peak (Figures 2, 2, 4). The retrograde tracer deposit was located ventrally in the nucleus (Figure 7C) and this explains the lack of retrograde labeling in S1 cortex in this case. In all cases shown in Figure 7, retrogradely labeled cells were located with equal density in the barrel and septal compartments (data not shown).

### Laminar Origin of Corticofugal Projections

**Figure 8:**
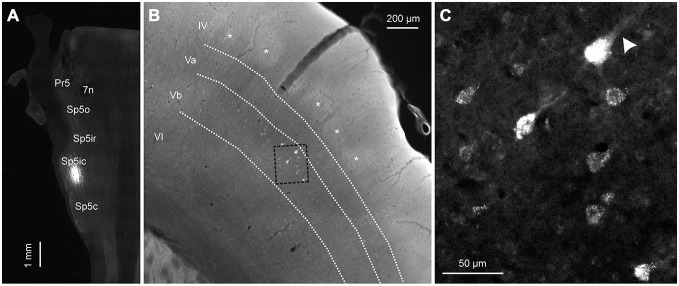
**A representative example showing the laminar distribution of retrogradely labeled cells in cortex following a tracer deposit into the Sp5ic. (A)** Horizontal section through the brainstem showing the location of the injection site in Sp5ic. **(B)** Coronal section through cortex showing the layer IV barrels (asterisks) in relation to the retrogradely labeled cells. The layer boundaries have been drawn from neighboring sections stained for CO and superimposed on this section. **(C)** Inset from **(B)**, shown at higher magnification, showing the retrogradely labeled cells with the presence of apical dendrites (arrowhead).

To determine the laminar origin of these corticofugal projections, two rats were injected with FG in the Sp5ic. In these cases, S1 and S2 were sectioned coronally instead of tangentially (Figure 8). As illustrated in Figure 8B, the retrogradely labeled cells reside in infragranular layer Vb. When comparing the position of the retrogradely labeled cells with the CO barrels, seen as lightly stained “ghost” structures in layer IV, it can be seen once again that the retrogradely labeled cells fall within both barrel and septal columns. The retrogradely labeled cells appeared to be large pyramidal neurons (Figure 8C) with an apical dendrite coursing towards the superficial layers (arrowhead).

### Absence of Projections from Motor Cortex

**Figure 9:**
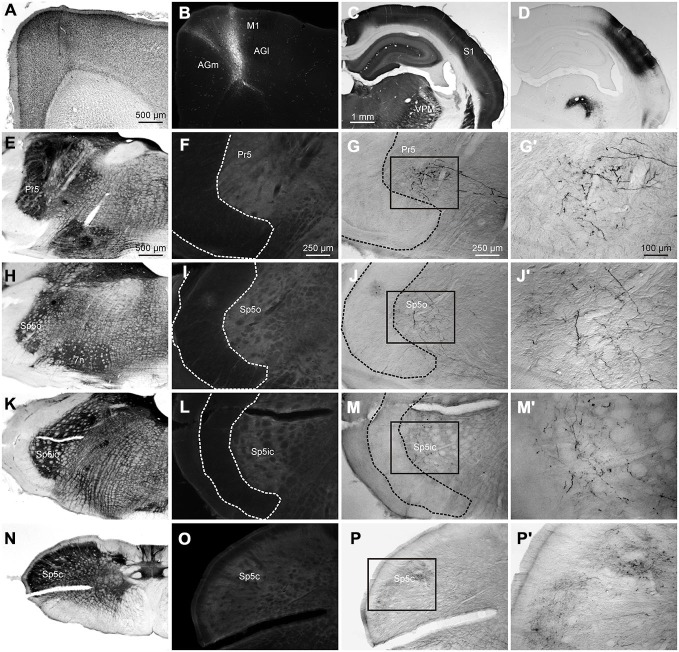
**A representative example of a case receiving dual anterograde tracer deposits into the M1 and S1 cortices. (A)** Nissl stained coronal section through the cortex showing the track of the pipette used to inject FR into M1. **(B)** Injection site of FR into vibrissal representation of the M1 cortex. **(C)** Coronal section through S1 barrel cortex processed for CO labeling. **(D)** Adjacent section, processed for visualization of BDA showing injection site in S1 and associated anterograde labeling. Anterograde labeling can be seen in ventral posterior medial nucleus (VPM). **(E)** Coronal section through the brainstem at the level of Pr5 stained for CO for better visualization of nuclear boundaries and neural tracts. **(F)** Adjacent section processed for visualization of FR terminals showing lack of any labeled terminals. **(G)** Adjacent section processed for visualization of BDA labeled terminals showing the presence of labeled varicosities in Pr5. **(G′)** Inset from **(G)** shown at greater magnification. **(H–J′)** Identical photomicrographs from the brainstem at the rostrocaudal level of Sp5o. **(K–M′)** Sections from the level of Sp5ic. **(N–P′)** Sections from the level of Sp5c.

In the retrograde tracing experiments, irrespective of the rostro-caudal location of the tracer deposit in the brainstem, we did not see any labeled cells in M1 cortex. Although this has been mentioned in the literature before (Miyashita et al., 1994; Desbois et al., 1999; Urbain and Deschênes, 2007; Alloway et al., 2010), we sought to confirm this by analyzing data from three animals from an earlier study which had received anterograde tracer deposits in M1 and S1. Figure 9 illustrates corticofugal projections from M1 and S1 to the trigeminal sensory nuclei whisker representations. In this case, a FR tracer deposit was made in the M1 whisker representation (Figures 9A,B), and BDA was placed in S1 barrel cortex (Figures 9C,D). Labeling in the ventral posterior medial nucleus (VPM) and posteromedial nucleus (POm) in thalamus confirmed that the tracer had been deposited in the S1 barrel field (Figure 9D). S1 corticofugal projections, as shown before, targeted mainly Pr5, Sp5ic and dorsal Sp5c (Figures 9G,J,M,P) whereas M1 corticofugal projections could not be seen in any of the trigeminal nuclei (Figures 9F,I,L,O). M1 labeling was however observed in other mescencephalic and brainstem structures such as the superior colliculus, the periaqueductal gray, the basal pons, the deep mescencephalic nucleus, the interstitial nucleus of the medial longitudinal fasciculus and the gigantocellular, parvocellular and intermediate reticular nuclei, consistent with previous reports (Hattox et al., 2002; Takatoh et al., 2013; Sreenivasan et al., 2014). For a detailed analysis of M1 projections to brainstem readers are referred to a previous study (Alloway et al., 2010).

## Discussion

In this study, we quantified the corticofugal projections to the trigeminal sensory nuclei by combining anterograde and retrograde tracing approaches. Apart from quantifying the number of labeled terminals from S1, S2 and M1 cortical injections in the different trigeminal sensory nuclei, we also determined the three dimensional labeling pattern of these terminals across rostro-caudal and dorsoventral axes of these nuclei. Further, retrograde tracer deposits at specific rostro-caudal positions along the chain of trigeminal sensory nuclei confirmed the projection patterns observed using anterograde tracers, and revealed the spatial pattern of these projection neurons in S1 and S2.

We found an overall similarity between the innervation patterns of S1 and S2 corticofugal projections with a few notable and important differences. S1 and S2 projections innervated all the sensory trigeminal nuclei with largely overlapping terminal fields. However, projections from S1 barrel cortex terminated mainly in Pr5, Sp5ic and dorsal Sp5c. The terminals were clustered in specific foci with sparse labeling in the intervening areas. By contrast, S2 projected across all trigeminal sensory nuclei and did not exhibit the patchy labeling patterns seen with S1 injections. Sp5c exhibited a dorsoventral gradient with S1 projections targeting the dorsal regions of this subnucleus, whereas S2 projections terminated throughout the entire dorsoventral extent. The S1 projection neurons were equally distributed throughout the barrel and septal columns and were situated in the infragranular layers, specifically in layer Vb. Finally there was a complete absence of projections from vibrissal M1 cortex to any of the sensory trigeminal nuclei.

Our data also show no clear spatial segregation of S1 and S2 projections in the trigeminal nuclei believed to play a critical role in sensory gating of the lemniscal pathway, i.e., Sp5ic and Sp5c (Furuta et al., 2010). This raises some doubt about the view that trigeminal gating is achieved by the complementary action of corticofugal projections from S2 and S1 on Sp5ic and Sp5c, respectively.

### Corticofugal Projections and Trigeminal Sensory Gating

A recent study suggested a possible explanation for the differences between active and passive sensory responses observed throughout the ascending lemniscal pathway (Furuta et al., 2010). The authors measured the activity of neurons in the trigeminal sensory nuclei in response to cortical electrical stimulation. Electrical stimulation of S2 activated Sp5ic interneurons whereas M1 electrical stimulation, which presumably antidromically activated neurons in the S1 septal columns, inhibited them. This inhibition was reduced upon lesioning Sp5c. The authors concluded that S1 septal projections to Sp5c activate GABAergic projections from Sp5c to Sp5ic, inhibiting the latter and releasing the neurons in Pr5 from their inhibition. This is thought to occur during passive whisker stimulation resulting in tactile responses that have a larger amplitude than those occurring during active whisking. For sensory flow during active whisking, the hypothesis holds that S2 projections to Sp5ic activate GABAergic projections that inhibit Pr5 neurons and reduce the amplitude of their sensory responses. Hence, gating of ascending sensory information from Pr5 would require complementary actions of S1 and S2 projections on Sp5c and Sp5ic, respectively. However, our data show that projections from S1 and S2 terminate in overlapping parts of Sp5ic and Sp5c. Further, we found no difference between barrel and septal projections to the Sp5ic and Sp5c, and this raises additional skepticism about the assertion that neurons in the S1 septal columns specifically control the Sp5c-mediated inhibition of Sp5ic. Equal distributions of retrogradely labeled cells in the septal and barrel columns of S1 also seems to contradict a recent report suggesting that S1 projections to Pr5 and Sp5c arise mainly from the barrel columns (Malmierca et al., 2014). We did not make any tracer injections into Pr5 for this study and thus can neither confirm nor deny the findings by Malmierca et al. (2014), nor compare them to our current data. If barrel columns do preferentially innervate Pr5, whereas both barrel and septal regions innervate Sp5ic and Sp5c, this may provide insight into the function of the corticofugal projections to these different subnuclei.

Our data therefore cast doubt on the idea that the synaptic connections of S1 and S2 could differentially affect the Sp5ic interneurons and thereby signal flow in Pr5. Although we question the specific mechanism proposed by Furuta et al. (2010) for sensory gating during active whisking, we do not doubt that both intra trigeminal interneurons and corticofugal projections could play crucial roles in such gating. A study conducted in awake rats has shown that lesioning the Sp5i nucleus reversed the effects of such sensory gating in the lemniscal pathway (Lee et al., 2008). This makes it very likely that Sp5i interneurons are indeed involved in modulating the lemniscal gate by their inhibitory action upon Pr5. The neurons in Sp5ic receive almost all of their input from three sources, primary afferents from the trigeminal ganglion, inhibitory projections from other trigeminal nuclei and descending cortical projections (Jacquin et al., 1986b, 1988, 1990; Furuta et al., 2006, 2008, 2010). The cortical projections are the most likely candidates for conveying information about active and passive states as the switch from passive to active states likely involves a cortical signal (Matyas et al., 2010). Given our data, S1 or S2 cortex alone could achieve this by direct projections to Sp5ic and Sp5c. Our data also allow for a direct influence of S1 and S2 on Pr5 via their direct projections there. Most likely these projections activate Pr5 neurons because inhibitory interneurons in Pr5 are extremely sparse (Avendaño et al., 2005; Furuta et al., 2008). To parse out possible mechanisms of sensory gating, electrophysiological recordings need to be performed within the brainstem of rats engaging in active and passive touches.

Additionally, although S1 and S2 project to both Sp5ic and Sp5c in overlapping areas, it is conceivable that S1 projections specifically synapse onto multi-whisker projection neurons in Sp5ic whereas the S2 projections innervate the mono-whisker interneurons. This would support the complementary sensory gating hypothesis although it still fails to explain the role of S2 projections to the SP5c. Such specific innervation of different neuronal populations in Sp5ic by the two cortical regions, although possible, is unlikely given the spatial overlap between the corticofugal terminals from S1 and S2. Clearly, further experimental evidence is needed to address this issue.

Finally, it is has been shown that S1 electrical stimulation facilitates sensory responses of neurons in Sp5ic when the receptive fields of the cortical and trigeminal neurons overlap, but are suppressed when not overlapping (Woolston et al., 1983). In fact, discrete corticofugal terminal patches from S1 and S2 injections that were seen most prominently in Pr5 may reflect such a topographical control exerted differentially by these cortical areas on this and possibly other trigeminal nuclei as reported previously (Malmierca et al., 2014). Therefore, corticofugal projections from a particular site may enhance sensory flow in trigeminal nuclei with similar receptive fields while inhibiting spatially dissimilar neurons with dissimilar receptive fields. Such additional inhibitory mechanisms may further complicate the interactions of S1 and S2 on trigeminal nuclei. Future studies should investigate the interaction of receptive field properties on S1 and S2 corticofugal interactions.

As mentioned earlier, ascending sensory information from the whisker pad is also conveyed via the extralemniscal and paralemniscal systems in addition to the two lemniscal pathways that have been described so far (Lo et al., 1999; Veinante and Deschênes, 1999; Pierret et al., 2000; Veinante et al., 2000b; Furuta et al., 2009; Wimmer et al., 2010; Ohno et al., 2012). Although we specifically address the hypothesis of sensory gating by corticofugal projections in the lemniscal system, it is important to note that the modulation of sensory information in the other pathways, their interaction with the lemniscal pathway and their dependence on corticofugal projections is unknown. However, given similar overlaps between S1 and S2 terminals across the different trigeminal sensory nuclei, it is plausible that similar mechanisms would operate on the other pathways as well.

### Additional Roles of Corticofugal Projections

Apart from the gating of sensory information, the corticofugal projections to trigeminal sensory nuclei could also have other potential functions, specifically in the processing of pain. The Sp5c is an important component of the trigeminal nociceptive pathway (Hu, 1990; Bereiter et al., 2000; DaSilva et al., 2002; Sessle, 2006; Okubo et al., 2013) conveying ascending information about oral and facial pain (Takemura et al., 2006; Weigelt et al., 2010). It has been, in fact, previously suggested that the corticofugal projections to Sp5c might play a role in nociceptive transmission during chronic pain such as in central pain syndrome (Malmierca et al., 2012, 2014). It is interesting to note that previous studies have shown that neurons responding to noxious stimuli are located specifically in dorsal Sp5c in laminae I and II (Renehan et al., 1986) where, according to our data, S1 projections terminate. A potential role in nociceptive processing for S1 is not unlikely given previous studies showing that S1 neurons show increased activity during the cortical generation of pain (Quiton et al., 2010).

### Technical Considerations and Challenges

It is important to note that dorsoventral differences in labeling in Sp5c could, in principle, originate from injecting different whisker rows in S1 and S2. The corticofugal projections from S1 and S2 are topographically organized (Jacquin et al., 1990) and the different rows are oriented dorso-ventrally in the trigeminal nuclei with row E located dorsally and row A ventrally (Arvidsson, 1982; Jacquin et al., 1986a, 1993). However, we consider this unlikely as, first, care was taken to inject corresponding whisker rows in S1 and S2, and second, retrograde labeling (which likely encroached many barellettes) confirmed our anterograde results. Also, since the rows are similarly oriented across the entire rostrocaudal length of the trigeminal nuclei, similar dorso-ventral gradients would have been expected throughout the trigeminal sub-nuclei. This, however, was not the case indicating that shifted injection sites within the whisker maps were not the basis of the differences observed.

## Funding

This work was supported by the DFG (Deutsche Forschungsgemeinschaft grant CH 1232/1-1 awarded to SC) and the NIH (National Institutes of Health grant 37532 awarded to KDA). The open access fee for this article was funded by the Open Access Publishing Fund of the University of Tübingen.

## Conflict of Interest Statement

The authors declare that the research was conducted in the absence of any commercial or financial relationships that could be construed as a potential conflict of interest.
